# Understanding health-seeking and adherence to treatment by patients with esophageal cancer at the Uganda cancer Institute: a qualitative study

**DOI:** 10.1186/s12913-021-06163-3

**Published:** 2021-02-18

**Authors:** Nakimuli Esther, Ssentongo Julius, Mwaka Amos Deogratius

**Affiliations:** 1 Department of Research and Training, Uganda Cancer Institute, Upper Mulago Hill Road, Kampala, Uganda; 2 Department of Disease Control & Environmental Health, School of Public Health, College of Health Sciences, Makerere University, Kampala, Uganda; 3 Department of Medicine, School of Medicine, College of Health Sciences, Makerere University, Kampala, Uganda

**Keywords:** Esophageal cancer, Health-seeking, Adherence to treatment, Access to care, Uganda

## Abstract

**Background:**

In the low- and middle-income countries, most patients with esophageal cancer present with advanced stage disease and experience poor survival. There is inadequate understanding of the factors that influence decisions to and actual health-seeking, and adherence to treatment regimens among esophageal cancer patients in Uganda, yet this knowledge is critical in informing interventions to promote prompt health-seeking, diagnosis at early stage and access to appropriate cancer therapy to improve survival. We explored health-seeking experiences and adherence to treatment among esophageal cancer patients attending the Uganda Cancer Institute.

**Methods:**

We conducted an interview based qualitative study at the Uganda Cancer Institute (UCI). Participants included patients with established histology diagnosis of esophageal cancer and healthcare professionals involved in the care of these patients. We used purposive sampling approach to select study participants. In-depth and key informant interviews were used in data collection. Data collection was conducted till point of data saturation was reached. Thematic content analysis approach was used in data analyses and interpretations. Themes and subthemes were identified deductively.

**Results:**

Sixteen patients and 17 healthcare professionals were included in the study. Delayed health-seeking and poor adherence to treatment were related to (i) emotional and psychosocial factors including stress of cancer diagnosis, stigma related to esophageal cancer symptoms, and fear of loss of jobs and livelihood, (ii) limited knowledge and recognition of esophageal cancer symptoms by both patients and primary healthcare professionals, and (iii) limited access to specialized cancer care, mainly because of long distance to the facility and associated high transport cost. Patients were generally enthused with patient – provider relationships at the UCI. While inadequate communication and some degree of incivility were reported, majority of patients thought the healthcare professionals were empathetic and supportive.

**Conclusion:**

Health system and individual patient factors influence health-seeking for symptoms of esophageal cancer and adherence to treatment schedule for the disease. Interventions to improve access to and acceptability of esophageal cancer services, as well as increase public awareness of esophageal cancer risk factors and symptoms could lead to earlier diagnosis and potentially better survival from the disease in Uganda.

**Supplementary Information:**

The online version contains supplementary material available at 10.1186/s12913-021-06163-3.

## Background

Esophageal cancer ranked seventh in incidence with an estimated 572,000 new cases and sixth in mortality overall with 509,000 deaths in 2018 worldwide. The reasons for increased incidence of cases of esophageal cancers are multiple but generally reflect both aging and growth of the population, changes in lifestyles including smoking, alcohol intake, and obesity, as well as changes in the prevalence and distribution of the main risk factors for cancers in general [1–3]. Esophageal cancer is quite common in South East Asia and Eastern Africa. In Uganda, esophageal cancer ranks seventh and accounts for 7.8% of all cancer deaths [2]. The incidence of esophageal cancer is on the increase in Uganda. Data from the Kampala Cancer Registry (KCR) show that between 1991 and 2010, the incidence of esophageal cancer in both male and female was increasing by about 1.6 to 3.3% per annum [4]. The esophageal cancer risk ratio in Uganda and most of Africa (male: female) is about 2:1 [5, 6].

Treatments for esophageal cancer include surgery followed by chemotherapy and or chemo-radiation depending on the stage of the cancer and patients’ functional status [3]. In spite of the available treatment modalities for esophageal cancer, survival from the cancer is generally poor in both low and high-income countries. For example, of 151 esophageal cancer patients included for a survival analysis study in Uganda in 2005, the 5-year relative survival was only 4.5%. This survival rate was comparable to that of black esophageal cancer patients from the Surveillance, Epidemiology, and End Results (SEER) registry in the USA [7]. In the low-income countries, majority (78.8%) of patients with esophageal cancer present for diagnoses after long periods (> 30 days) with symptoms [8]. In particular, the majority (50–90%) of esophageal cancer patients in Africa present with advanced stage disease [8, 9], and often experience poor survival of not exceeding 12 months from diagnoses [10]. The reasons for long time to presentations and advanced stage at diagnoses are not well understood. Delayed health-seeking and or poor adherence to cancer specific treatment are two patient-related factors that may influence access to and remaining in care. Health-seeking behaviors for symptoms may be defined as the intentions, thoughts, decisions, and actions aimed at accessing healthcare services in response to symptoms being experienced or perceived problems that challenge personal abilities [11]. On the other hand, self-reported or indirect measure of adherence to treatment could be understood as the degree to which a cancer patient’s behavior corresponds with the agreed recommended course of treatment by taking all prescribed medications for the length of time necessary and attending hospital visits as scheduled [12–15]. Several factors influence health-seeking and adherence to cancer specific treatment. For example, there is evidence that esophageal cancer patients often have fears regarding surgery, chemotherapy and radiation because of perceived side effects [16]. In addition, patients’ individual factors including perception of treatment modalities influence their health-seeking decisions [17–19]. Patients’ treatment seeking behavior and adherence to treatment are also influenced by the challenges patients experience during the course of illness and treatment including stigma, psychosocial stress, and loss of employment due to the chronic nature of the disease and chronic absenteeism from work [20]. Among esophageal cancer patients, individual factors that have been shown to limit prompt health-seeking for esophageal cancer symptoms include age, education attainment, marital status, income, knowledge about the disease and attribution of symptoms [21]. Healthcare system factors that are associated with delayed health-seeking and advanced stage at diagnoses include longer distance to the health facility, inadequate information given by the healthcare professionals and delayed recognition of symptoms and referral [22]. In Uganda, esophageal cancer incidence is remarkably on the rise among both male and female and yet there is limited data on factors influencing health-seeking and adherence to treatment at the national cancer treatment center, the Uganda Cancer Institute (UCI). This study aimed to explore factors influencing esophageal cancer patients’ health-seeking behavior and adherence to treatment at the UCI, Kampala. Findings from this study can inform targeted awareness messages to patients and the healthcare providers to promote prompt health-seeking and adherence to cancer specific treatments at the specialized cancer treatment facility.

## Methods

### Study site

This study was conducted at the Uganda Cancer Institute, the only cancer specialized, tertiary public facility in Uganda. The facility is located in Kampala, Uganda. The Uganda Cancer Institute offers specialized cancer treatment, research, and training [23].

### Study design

This was an exploratory interview based qualitative research. A qualitative approach was adopted because it has potentials to provide an in-depth understanding of factors that influence esophageal cancer health-seeking and adherence to treatment schedules [24, 25]. This detailed insight can potentially inform design of interventions to improve esophageal cancer health-seeking and treatment services provision in Uganda and other low-income countries.

### Study population and inclusion criteria

Patients with confirmed diagnosis of esophageal cancer at any stage, and aged 40–80 years attending care at UCI participated in the in-depth interviews (IDIs) during the study period. This age group was considered because records from UCI and literature showed they are the category most at risk of esophageal cancer [26, 27]. Healthcare providers including doctors, nurses, counselors and social workers who had direct contact with esophageal cancer patients in care participated in this study as key informants. Other inclusion criteria were ability of patients to respond to the study questions and provisions of a written informed consent. Patients were excluded if they were considered very sick and unable to talk due to complications of the disease or other documented reasons for aphasia/dysphasia. Healthcare professionals involved in care of patients with esophageal cancer but who were not available at the facility during the study period for any reasons were excluded. There was only one such healthcare professional; she was on annual leave and could not be reached during the study period.

### Sample size and sampling techniques

Sample size was determined by the data saturation approach. We conducted in-depth interviews with patients and healthcare professionals until when two or more additional interviews would not add any new view points, i.e. the point of data saturation [28, 29]. We included 16 patients and 17 healthcare professionals in this study. We used non-probability purposive sampling methods to select participants for interviews. We included both outpatients and inpatients. Patients were recruited at the outpatient gastro-intestinal oncology clinic and solid tumor ward of the UCI. To ensure a diverse sample, patients were selected purposively ensuring we capture patients with a range of characteristics including stage of disease, age and sex. For the healthcare professionals, selection was based on those who were taking care of patients with cancer of esophagus during the study period.

### Recruitment and data collection

The first author and two trained research assistants recruited participants and conducted interviews. The research assistants were trained for 2 days on the study purpose, objectives, sampling and recruitment procedures, consenting, and qualitative interviewing techniques and data transcription. Data were collected using pretested interview guides developed based on literature regarding patients’ experiences of esophageal cancer symptoms and help-seeking. The interview guides were pretested in a nearby faith–based hospital, St. Francis hospital, Nsambya (about 5 km to the south of UCI), that provides specialized cancer care. Data from the pretest were analyzed manually to gain insights into the themes related to our research objectives. The interview guide/tool was consequently refined to capture the relevant issues related to this study objectives (Additional files 1 & 2). The interview guide for patients was translated to Luganda, the most commonly spoken local language in Kampala and then back translated into English to ensure accuracy and consistency. The two versions were reviewed and adjusted accordingly. Both the English and Luganda versions were used in the study depending on patient’s preference. We collected data during July and August 2019.

Ethical approval and permission to conduct the study was obtained from the Makerere University School of Public Health Higher Degrees, Research and Ethics Committee. Institutional clearance for conducting the study was sought from the Director of research at the Uganda Cancer Institute. Individual written informed consent was obtained from every participants before interviews were conducted. We approached patients individually after obtaining a list of registered esophageal cancer patients from the UCI record department, explained to them the study purpose and what it involved. Patients who accepted to participate in the study were taken to a quiet, comfortable and convenient room within the facility for further explanations and consenting. Adequate information was provided to the prospective participants regarding the study. Prospective participants were assured of privacy and confidentiality regarding use of data obtained. They were also informed that participation is voluntary; that they can start to participate and withdraw at any time, and that their refusal or withdrawal would not attract any negative consequences regarding their care at the UCI. Participants provided written informed consents before participation in the study. The trained research assistants and first author conducted the interviews. Each interview lasted about 30 to 50 min. Interviews were audio-recorded with consent from each participant.

The healthcare professionals were recruited based on a list obtained from the Clinical Director and Nursing Officer in charge of the Gastro-enterology Department of the UCI. Only healthcare professionals directly involved in the care of esophageal cancer patients were included. There were six doctors, six nurses, three clinical counselors, and two social workers. We approached these healthcare professionals individually and explained to them the study purpose, objectives and rationale. Those who demonstrated interest to participate received additional information on consenting and data collection procedures. No healthcare professional declined participation. Scheduled interviews were conducted in person after provisions of written informed consents. All interviews were audio-recorded, and lasted 35 to 60 min.

### Data management and analysis

The first author and research assistants transcribed the interview recordings verbatim. Handwritten notes taken during interviews were used to enhance data during transcriptions. Interviews conducted in the local language were translated during transcriptions. The first author participated in all the transcriptions and translations of the interview recordings. Interview transcripts were shared between the investigators who read through them independently to gain insights into the data before formulation of codes for data analysis. Each investigator developed codes. The three investigators discussed, and agreed on the final codes for analyses through consensus. The final set of codes constituted the codebook used in data analysis. Manual data analysis using the thematic content analysis approach was used [30, 31]. The first author applied the codes on every meaning segments of each transcript, reading through the transcript line by line. The coded segments with similar meanings were aggregated to form subthemes and themes that reflected their central meaning. The analysis framework was shared between the investigators who discussed and agreed on the themes and subthemes. Further analysis was informed by the data and involved iterative coding and aggregating meaning segments from each transcript. Recurrent themes formed the main framework for data interpretation based on the main outcome variables of health-seeking for symptoms of esophageal cancer and adherence to recommended esophageal cancer treatment.

Participants’ socio-demographic variables including sex, age, marital status, education attainment, and duration of work as healthcare professional, and patients’ experiences right from first symptoms realization and attributions, help-seeking, and diagnosis as well as perceived and manifest stigma, stress, and threat to or actual loss of employment guided contextual data interpretation under the themes and subthemes. We did not aim to compare and contrast views from the patients and healthcare professionals, but rather identify common issues that potentially influenced health-seeking and adherence from the perspectives of the two categories of participants.

## Results

### Participant characteristics

Majority of the patient participants were male (62%) with median age of 50 years. Most participants resided in the rural areas (56%) and were married (50%) (Table 1).

**Table 1:** Socio-demographic characteristics of patient participants

Characteristics ***N*** = 16	Frequency	Percentage
**Sex**		
Female	6	37.5
Male	10	62.5
**Residential status**		
Rural	9	56.3
Urban	7	43.7
**Education status**		
Primary or none	11	68.8
Secondary	5	31.2
Tertiary	0	0.0
**Marital status**		
Single	4	25.0
Married	8	50.0
Divorced	2	12.5
Widowed	2	12.5
**Age (years)**		
Median age, Overall = 50; female = 63; male = 55. Range = 40–79		
40–49	3	18.8
50–59	6	37.5
60–69	6	37.5
70–79	1	6.2
**Current occupation (surrogate measure for income status)**		
Not indicated	2	12.5
Peasant	9	56.2
Self employed	3	18.8
Formally employed	2	12.5
**Region of residence**		
Central	8	50.0
Eastern	3	18.8
Western	2	12.4
Southern	0	0.0
Northern	3	18.8
**Stage at Diagnosis**		
Stage I/II	0	0.0
Stage III	2	12.5
Stage IV	14	87.5

The healthcare professional participants included both male and female, and were doctors, nurses, and social workers. However, the counselors were only female (Table 2).

**Table 2:** Distribution of healthcare professionals (key informants) by sex and designation

Sex	Designation	Number
**Female (*N*** **= 10)**	Doctors	3
	Nurses	3
	Counselors	3
	Social workers	1
**Male (*N*** **= 7)**	Doctors	3
	Nurses	3
	Counselors	0
	Social workers	1

### Main themes

We identified four main themes related to health-seeking and adherence to treatment: 1) emotional and psychosocial factors, 2) limited knowledge and recognition of esophageal cancer, 3) limited access to specialized cancer care, and 4) patient-provider relationships and communications. Verbatim quotes representing the main message under each subtheme have been included in the results to validate the themes. The quotes are identified by; designation of healthcare professionals, age categories of patients, and sex of participants. Throughout the results, we have referred to the patient participants as patients, and the healthcare professional participants as healthcare professionals.

### Emotional and psychosocial factors

Patients presented varying experiences related to their help-seeking journeys that often started with realization of abnormal bodily changes related to swallowing food and or water. These were followed with appraisal and attribution of possible causes of the symptoms. Several emotional and psychosocial factors emerged from the data that are related to the health-seeking intervals as well as the extent of adherence to treatment regimen. These factors included stress and stigma associated with knowledge of a cancer diagnosis, and fear of loss of livelihoods as patients take time away from employments and farm activities while seeking care and receiving treatments for the cancers. In the context of this study, we have considered the concepts of stress, distress, fear and anxiety collectively because we are mainly concerned with their effects on help-seeking decisions and actions, and on adherence.

#### Stress related to cancer diagnosis

Majority of patients reported experiencing a high degree of stress and distress when they learnt about the diagnosis of cancer; they subsequently developed fear, lost hope, and experienced compromised quality of life. We found that the journey to the specialized cancer treatment center was challenging as patients often made several visits before getting the expected help.

*“When I was told that I had cancer of the throat, I got a lot of fear; I faded, I lost weight, and I felt so bad. […]Then when they referred me to UCI, I could come several times but without getting any help from them. This stressed me so much and many times I felt I should just give up”* (Female, 60 – 69 years)

Similarly, the healthcare professionals affirmed that esophageal cancer patients undergo several challenges and experience a lot of stress that could influence treatment seeking and adherence to treatment. They pointed out that knowledge of diagnosis was like a heart break, the treatment itself is troublesome, and that patients’ lives after esophageal cancer diagnosis is generally stressful. This view cut across the different categories of healthcare professionals included in the study.

*“Of course, everyone with esophageal cancer is stressed; remember when you hear cancer, they come with mixed feelings. Others fear they are going to die, and leave their young ones. Others fear to lose their marriages and it has happened as a partner who was not sick abandoned a person who is sick because they have heard of cancer. You know they don’t know what it means; they think it is infectious, they are going to get it from them; so, they just abandon them.”*(Female, Counselor).

#### Stigma related to esophageal cancer diagnosis

Patients reported experiencing stigma when friends and relatives got to know of their cancer diagnoses. The stigma were related to the weight loss brought about by the cancer. The stigma made patients feel rejected, unwanted and miserable. As a result, majority of patients reported developing self-hatred, feelings of worthlessness and hopelessness. They also reported thoughts of withdrawing from treatment. Some patients reported developing suicidal ideas because of the feelings of rejection by the community.

*“People talk! Some understand and they could encourage me but others, like there is one whom I considered the best friend. One time she told me that she could not go to people when she has lost weight. […]. Then another time this very lady asked me that eh Nnalongo (mother of twins) as you have lost much weight do you want to follow your mother? Imagine my mother had just died three months ago. […]. Then another time I found her at the mobile money and told her that eh this time the throat is over paining me; then she told me that why don’t you die and we eat rice; these are four times this lady is making me feel bad”* (Female, 70-79 years).

Most of the healthcare professionals concurred that patients with esophageal cancer experience stigma from the community mainly because of the massive loss of weight, vomiting, coughing and the bad breathe that come with the disease. The society often detests these symptoms especially bad breathes, and they shun the patients. The healthcare professionals reported that members of the public often avoided patients with bad breathes and made unfavorable comments about them.

*“Even other people when they are travelling, they find them with bad smells and get out of the vehicles! And what do you expect the driver to do? He instructs the patient to disembark and that is already stigma. So eventually of course he will not come. They will abscond and they will decide to stay home with their bad odors other than inconveniencing other people. Some of them or most of them are suicidal; they have suicidal tendencies. He feels like if this is the case - people are fearing me, I have no finance to facilitate treatment, I’m in pain myself - why don’t I die”* (Female, Nurse).

#### Fear of loss of livelihood

Most patients reported threat to their jobs and sometimes actual loss of employment during the course of their illnesses. Patients require time off work to undergo treatments that take several months. Majority of the participants reported experiencing severe side effects and several episodes of hospitalization when undergoing treatment. They reported that most of the employers could not tolerate the extended period of absence from work, and they ended up relieving cancer patients of their duties. The participants reported the loss of employment would affect their treatment adherence due to lack of money to pay for health services, transport and home needs.

*“Umm, you see because of this sickness, I was terminated from work and now I am surviving on God’s mercy. I was working as an accountant in Lacan (not real name) Bank. But because of the side effects of chemotherapy, I got so weak that I could no longer go to work. I asked for a leave but still I wasn’t any better by the time the leave ended. So, my supervisor said they could not extend my leave and so someone else had to occupy my position. They ended up terminating my contract. I was not paid for six months and the next two months they were like go off. And here I am now, I cannot even afford transport to come to the hospital; I just depend on other people. I think it’s really hard for someone to continue with normal work while battling with this disease”* (Male, 40-49 years)*.*

Similarly, participants without formal employment also reported experiencing extreme difficulties supporting themselves during treatment as they were often very weak due to treatment side effects and would be away from their gardens – the main way they often generated money for their livelihood.

*I’m a peasant but now I cannot dig; even my food got spoilt from the garden because I could not go there anymore”* (Male, 50-59 years).

All the healthcare professionals reaffirmed that loss of employment is a common challenge among esophageal cancer patients. They attributed the loss of jobs to repeated absence of the patients from work for so long a time for their employers to tolerate. They said the loss of employment often affected the patients’ treatment adherence because of the subsequent lack of money for transport to the hospitals and for payment of investigations and other services not available at the UCI.

*“Definitely as I have told you there was one who was a driver; eventually he could not drive so he was terminated. That even enhanced his sickness because he could not have now finance to manage his sickness, transport costs. He defaulted treatment, and buying the drugs which are out of stock; even eating. Of course when you don’t have food, how can you take in medicine” (*Male, Social worker).

### Limited knowledge and recognition of esophageal cancer symptoms

There was generally limited knowledge on esophageal cancer and poor recognition of its symptoms by the patients. This affected their appraisal and attribution of the cancer symptoms, and subsequently influenced their health-seeking, often delaying appropriate diagnosis and treatment. The healthcare professionals reported that healthcare professionals at the lower level facilities (primary healthcare professionals) often have low knowledge regarding esophageal cancer risk factors, symptoms, and appropriate treatment. This limitation of the primary healthcare professions affect the promptness of diagnosis and referral of patients with esophageal cancer symptoms to the specialized centers for histology diagnosis and treatment.

#### Low awareness of esophageal cancer disease

Majority of the patients reported that when they started experiencing the symptoms of esophageal cancer, they took it as a minor illness and just kept using pain killers to soothe the pain. Most of the patients were unaware of the risk factors for esophageal cancer and did not think about the cancer when they experienced symptoms.

*“For sure I have no idea what causes this disease, but they say smoking and drinking alcohol can cause cancer. I don’t know whether it’s what caused my cancer . . . I consumed alcohol and cigarette for over 15 years*.*”*(Male, 50-59 years).

The healthcare professionals reported that most esophageal cancer patients they cared for had very limited awareness of the disease. They thought that the patients often sought care inappropriately because of their limited awareness of esophageal cancer symptoms.

*“Most time patients may think it is another type of cough . . . so they end up treating it as a minor illness. So, because both patients and health workers do not know about esophageal cancer, patients are always referred at Cancer Institute when the disease is already advanced”,* (Male, Nurse).

Primary healthcare professionals were also reported to have limited knowledge of esophageal cancer. The patients revealed that their primary healthcare professionals kept treating symptoms as peptic ulcers or some other minor illnesses.

*“Yes there were barriers in trying to get the right diagnosis; because instead of checking for the right disease, most doctors in the health facilities I went to were just treating ulcers. And others could not tell me the kind of disease they were treating; it means even them, they did not know the symptoms of the disease and they were just gambling. Otherwise if they had told me to do the right tests for the disease, probably it would have been discovered earlier and something could have been done in time”* (Male, 50-59 years).

The perceived low awareness of esophageal cancer among primary healthcare professionals was shared by the healthcare professionals in this study; they concurred that the primary healthcare professionals lack adequate knowledge about esophageal cancer and so are not able to recognize the symptoms of this disease, especially when in the early stages. Therefore, these primary healthcare professionals end up misdiagnosing and mismanaging patients, and they think of referral when the disease has advanced.

“*The primary healthcare givers; it seems cancer does not ring in their minds immediately when patients come to them. In the first place, the patients begin by going to drug shops to buy pain killers when there is pain. May be when the situation is worsening they go to a clinic to see the clinicians there, a medical doctor or even a nurse but these will not think about cancer. They will start by gambling treating other things. Then later with time the disease could be growing by the time they realize, it is something bigger than what they have been thinking; it is already late”* (Female, Social worker).

#### Lay consultation and symptoms attribution

Most patients first consulted with friends and relatives, and then the traditional healers. They used the traditional and alternative medicines for varying length of time. Some patients resorted to traditional and alternative medicine when they had not experienced expected response from biomedical care provided by primary healthcare professionals. A minority of patients sought care with traditional and alternative medicine providers after histological cancer diagnoses.

*“I told my fellow soldiers in the barracks who advised me to go for treatment. When they treated me for some time and the condition was just worsening, they wrote for me a letter to leave barracks and go home. In the barracks they told me that this disease was not medical may be someone had given me poison or I had been bewitched. So I was sent home so that I can try other alternatives”* (Male, 50-59 years).

Likewise, almost all the healthcare professionals in this study reported that patients’ attributions and perceptions of symptoms explain in parts late presentation of esophageal cancer patients for care and the non-adherence to treatment for esophageal cancer. They asserted that most times patients attributed the symptoms to witchcraft, so instead of seeking medical care, they resorted to alternative treatment options like going to traditional healers, and prayers and worships. They would only seek medical care after spending a long time with the witchdoctors, spiritual healers, and the traditional and alternative medicine healers when the disease is already in advanced stages.

“*Oh yes; another reason they come late is trying alternative treatments like witchcraft, herbal medicine and prayers. They think of being bewitched, so they first go and waste time and even money in witchcraft while the witchdoctors play on their psychology saying somebody has bewitched them. Until one day someone opens up their mind and then they go for medical checkup! But most of them if they take a pain killer and it is not working they will go for those traditional doctors … All those are reasons why these patients come late”* (Female, Counselor).

### Limited access to the specialized cancer care facility

Access was perceived to be limited in terms of long distance to the specialized cancer facility, and affordability in terms of transport and medical requirements. Long distance to the specialized cancer facility was reported in almost all in-depth interviews to contribute to late presentation of patients for care as well as affecting adherence to treatment. Participants (patients) reported that it was very costly for them to come to the specialized cancer facility mainly in terms of transport and this was why they delay; they would meanwhile be looking for money. Further, treatment sometimes required prolonged hospital stay. Patients who did not have money for their hospital upkeeps postponed their scheduled treatment visits until when they had mobilized some funds, thus affecting their adherence to treatments.

*“Umm actually what I can say, I have a challenge of transport because if I’m to wait for army ambulance the process takes long. So, most of the time I transport myself and the costs are really high for me. Some time I run short of money to keep me at the hospital because the salary I get, I have to share it with the family so I find it gets finished and I’m left with nothing to come with at the hospital. Another thing is money for buying medicine, I can’t always wait for the army to buy me medicine, so I buy it sometimes and I find it very expensive”* (Male, 50-59 years).

Long distance and challenges meeting transport costs was reiterated by majority of the healthcare professionals. The healthcare professionals revealed that cancers advanced to late stages while patients would be looking for money for transport, medical requirements, and hospital upkeep.

“*Another thing is distance to the health facilities especially to the UCI; it really affects them because I have analyzed most of the patients we have here come from very far. This could be because the services here are free and they are people who are really very poor. The ones (patients) who are around Kampala can afford private facilities. So they are really not affected but the ones who come to Mulago are really affected by distance” (*Female, Doctor).

### Patient – provider relationships and communications

We found that the patient - doctor relationship and in particular, the perceived quality of communication between the patients and providers is important not only in the development and growth of the therapeutic alliance but also adherence to care plan. Inadequate information and guidance from healthcare professionals and perceived lack of empathy and courtesy were reported as factors that negatively influenced adherence to cancer therapies.

#### Inadequate communication about diagnosis and treatment plans

The majority of patients reported receiving only little information about esophageal cancer disease and treatment from healthcare professionals at the lower levels and UCI. They therefore would not know what to do, and what to expect regarding treatment plans and procedures. It was mentioned that esophageal cancer patients often wandered about and got information from fellow patients. Some of these information could be misleading, and could account in part for poor adherence to treatment.

*“No I don’t think they gave me enough information because, they only talked to me once and yet at that time my mind was not okay. I wish they could talk to us several times so that we get the information”, (*Female, 50-59 years).

Similarly, the healthcare professionals were in agreement that patients received little information about the disease, treatments and procedures. They attributed this irregularity to the overwhelming numbers of patients they attend to on a daily basis.

“*They do get information but at a minimum because of the overwhelming number of patients. The staffs do not really have enough time to sit with them; and at the same time we have only one health educator, about two social workers and three counselors. So those would be the people who have enough time to talk to them but the fact that they are few, they cannot also reach everyone”* (Male, Doctor).

#### Empathetic and respectful healthcare professionals

Majority of the patients perceived their providers as friendly, empathetic, courteous and caring. They reported that their providers handled them well compared to providers elsewhere. Only a few patients expressed some dissatisfaction; they reported that some healthcare professionals were constantly rude to them. These patients attributed the unbecoming behavior of these healthcare professionals to heavy workloads which may not allow them to keep calm all day long.

*“Well, the health workers here are not bad, only that whenever there is something good there is always something bad also. When you have got a problem, they are not people you can easily get. Sometimes you talk to them and someone responds rudely but not all. Again some are so friendly to us”* (Female, 50-59 years).

The healthcare professionals were also in agreement that the patient - provider relationship was amiable and supportive. They confessed that the healthcare professionals try their level best to handle their patients with respect. The healthcare professionals however acknowledged that there are a few instances of unintended misconducts towards the patients, especially by the nurses.

*“It depends; some patients are friendly to certain nurses and they talk ill about other nurses. So since we are human, we behave differently therefore it depends on how you handle a patient. They report that they talk to them rudely but most time it’s because a patient comes when you are very busy and they tell the patients to wait. They may not understand what you are telling them even if you are right; so the patients will just say the other nurse barked at me, mistreated me and so many things”* (Male, Nurse)

## Discussion

We found that health-seeking and adherence to esophageal cancer treatments are influenced by (i) emotional and psychosocial factors including stress of cancer diagnosis, stigma related to esophageal cancer symptoms, and fear of loss of livelihood related to loss of energy and disability from disease, and absenteeism from work, (ii) limited knowledge and recognition of esophageal cancer symptoms by both patients and primary healthcare professionals, (iii) limited access to specialized cancer care, mainly because of long distance to the specialized cancer care facility and associated high transport cost, and (iv) acceptable patient – provider relationships and communications at the UCI.

Emotional and psychosocial factors including stress of cancer diagnosis and stigma related to esophageal cancer symptoms and diagnosis influenced patients’ decisions and prolonged time to health-seeking. Adherence to cancer treatment regimens were also negatively affected by stigma; some patients were uncomfortable being seen in public including public transport means mainly because of the negative comments related to their excessive weight loss from poor feeding as a complication of esophageal cancer. Severe wasting have been associated with HIV/AIDS and curses [32]. Patients feel sad being associated with these conditions. They experienced social stigma, making them hide away. Foul smell from the mouths of esophageal cancer patients were some of the reasons for social exclusions. The patients reported that they could do nothing to stop the bad smell. They were aware of it; they saw people avoid them because of the smell. This made them feel sad and unwanted. Even in the public transport to the hospital, other passengers moved away from near them; people would not want to sit next to them. This scenarios made the patients feel bothersome to others, and sometimes they missed their treatments because they would not want to be sources of inconveniences to other people in the buses or taxis. The patients found themselves in awkward situations; travelling to the hospital meant inconveniencing other travelers, and not going to the hospital/UCI for specialized cancer treatments meant missing treatments and hence promoting cancer progression, worsening symptoms including pain and discomfort. An earlier study among breast cancer patients at the UCI revealed that perceived and internalized stigma interfered with treatment adherence and completions [33]. Self-worthlessness due to social and internalized stigma is common and very destructive to patients, leading to low self-esteem, poor treatment adherence and worse disease outcomes [34]. Stigma to HIV/AIDS symptom of wasting was a serious factor in poor adherence to treatment in Africa for decades. This led to high death rates among the HIV/AIDS population especially in the pre-antiretroviral therapy era [35–37]. Stigma to cancer symptoms and diagnoses have been reported among patients with other cancers including lung, colorectal, breast and cervical cancer [38–40]. Fighting stigma in health facilities is a critical step in improving treatment adherence, patients’ experiences in the facilities and recovery from illnesses. However, most health facilities do lack approaches aimed at reducing stigmas against patients at health facilities [41]. Formation of psychosocial and support groups have helped reduce stigma in clinical environment among patients with lung cancer [42]. More studies are needed to describe context specific, culture sensitive and cost effective approaches to stigma reduction among patients with cancers and other diseases at health facilities and the communities. These stigma reduction measures could lead to prompt health-seeking and better patients’ adherence to treatment and improved treatment outcomes.

In this study, concerns about loss of livelihood, and loss of employment due to prolonged hospitalizations and continuous absenteeism because of treatment were common experiences among patients. This impacted negatively on their treatment seeking behavior and adherence to treatment. Participants who lost their employments experienced more financial strains over the course of their treatments. Participants who were self-employed reported decline in their business returns and associated financial hardships. Patients reported that getting money for transport, investigations and treatments were a challenge they had been experiencing since diagnoses of the cancers. They depleted their financial resources within the first few visits to the cancer center. Their businesses declined while those in formal employments lost their jobs, narrowing further their financial bases. The patients reported that they became weak and frail following cancer diagnoses and treatments, and these undermined their physical abilities to carry on with their duties especially those engaged in works that required physical strengths including farming and construction works. The lack of health insurance to the general population in Uganda puts many patients at risk of catastrophic financial expenditures that subsequently undermine the functioning of their families. Cancer diagnoses have been associated with several challenges for both employees and employers in terms of work absenteeism and presenteeism [43]. Women with breast cancer in Canada experienced various challenges at work after diagnoses including unwanted changes in tasks, demotions, diminished physical ability, and job loss [44]. Loss of jobs and difficulties finding employments after cancer diagnoses is common all around the world and present a real challenge to cancer patients and their families [45–48]. Cancer diagnoses therefore come with various challenges regarding work and remunerations. Cancer workplace policies are needed to cater for cancer early detection through awareness but also for supportive mechanisms to reduce workplace stigma and secure patients’ jobs post cancer diagnoses.

Inadequate knowledge about esophageal cancer risk factors and symptoms reportedly delayed patients’ health-seeking. In symptomatic cancer patients, appropriate interpretation of symptoms plays an important role in timely diagnosis [49]. Majority of the patients reported low self-perceived risks for esophageal cancer and attributed symptoms to other mundane causes including ulcers. The patients reported that they could have presented for diagnoses at the specialized cancer center earlier if they knew that the symptoms they treated as other diseases were symptoms of esophageal cancer. The non-specific nature of symptoms of early stage esophageal cancer likely contributed to the misattributions. Our findings are similar to results from other studies which showed that patients often ignore or underrate the early symptoms of esophageal cancer which are often subtle and non-specific; they only seek care when the symptoms have worsened, at which point the cancer would be in advanced stages [50]. A questionnaire-based study in the UK that involved 96 esophageal cancer patients revealed that majority lacked adequate awareness of esophageal compared to breast cancer [51]. Unawareness of and or ignoring early cancer symptoms and or attributing them to comorbidities are common in many other cancers including breast cancer [52]. This has often resulted in delayed health-seeking and advanced stage disease at diagnoses [52–54]. Most esophageal cancer patients report a long interval between symptoms onset and presentation for diagnosis [55, 56]. It is critical that the population is made aware of the common risk factors and symptoms of esophageal cancer. This could help them in the determination of their own risks and aid appropriate interpretation of upper gastrointestinal symptoms. However, there are also data from a large retrospective cohort study in Italy showing that for esophageal cancer the time from first symptoms appearance to diagnosis and treatment seems to insignificantly influence treatment outcome and survival. That study also showed that longer time to diagnosis did not affect the choice of treatment type [57]. In another study from the Netherlands, it was showed that length of pre-hospital delay (from onset of symptoms until endoscopic diagnosis) did not affect patient’s short- or long-term outcome [54]. In spite of these data, a key principle for cancer control is to promote diagnoses in early stages. Promptness of diagnosis in order to reduce the incidence of advanced stage at diagnosis and to improve therapeutic efficacy is a key point in cancer control policies [58, 59]. In order to establish whether or not time from first symptoms occurrence to diagnoses are important in the case of esophageal cancer, more quantitative studies with accurate estimation of the point at which esophageal cancer symptoms have started are needed in order to determine the value of promptness of diagnosis based on symptoms presentations.

Esophageal cancer participants reported that their primary healthcare professionals who managed their symptoms before cancer diagnoses told them some other diagnoses including ulcers and gas in the stomach. They revealed that they were treated for these symptoms for needlessly long durations. Subsequently, they were referred or they self-referred themselves to the higher level health facility where endoscopies were done and the diagnoses of cancer were made. The healthcare professionals in the study also reported that patients come to the UCI when they have been managed for variable long durations for dyspepsia, gastro-esophageal reflux disorders (GERD), and peptic ulcer diseases (PUD). They contended that the diagnoses of a possible esophageal cancer was never on the mind of the primary healthcare professionals even in circumstances when the symptoms were very straightforward and typical of the disease. The healthcare professionals attributed the apparent low diagnostic acumens for esophageal cancer symptoms to multiple factors including inadequate training and insufficient continuous medical education (professional development) in Uganda. Low diagnostic skills and inability to recognize cancer symptoms by primary healthcare professionals have earlier been described among clinicians caring for cervical cancer patients in several sub Saharan African countries including Uganda and South Africa [60–62]. This study suggests the need for targeted educational interventions to increase the knowledge base and diagnostic acumens of primary healthcare professionals for esophageal cancer. If the primary healthcare professionals were knowledgeable about the disease, they could be able to give timely guidance, information, diagnosis, and referral.

High transport costs and long distances to the specialized cancer facility hindered patients’ promptness to seek care at the UCI and adhere to the treatment schedules. Participants reported that patients would postpone health-seeking visits and miss appointments for their treatments because of lack of money for transport and upkeep. The UCI is the main public specialized cancer treatment center in Uganda. It is located in the southern part of the country. Majority of patients from upcountry places therefore travel long distances of 200–400 km to reach the center. The public transport fare to Kampala of USD 10.0–20.0 is relatively high and unaffordable to majority of Ugandan patients from the rural areas where poverty is rife. A similar study [63] also found that distance to health facility negatively affected utilization of health services by cancer patients. The challenge of high transport costs to the cancer treatment center can be overcome by decentralization of specialized cancer treatment facilities to the regional referral hospitals that are closer to the patients and would require less transport money and travel time. Decentralization of services will perhaps reduce late presentation of patients and non-adherence to treatment regimens with consequent improvement in treatment outcomes and survival.

In this study, majority of the patient participants reported that their healthcare providers were generally empathetic and respectful although some nurses were accused of rudeness towards the patients. The key informants also reaffirmed that some healthcare providers at the UCI do not respond to patients’ questions appropriately and sometimes shouted at the patients, causing them to fear to inquire about their care plans. Poor or inadequate communications between patients and their clinicians has potential negative impacts on treatment adherence, and hence outcomes [64]. When patients perceive that the healthcare providers are friendly and welcoming, they are more likely to consult and remain in care. Patients and their families feel comfortable and may adhere to treatment plans when providers respond to patients’ questions with respect and openness. On the other hand, perceived or actual challenges in the patient - doctor relationship is likely to hamper health-seeking and adherence to treatment. In a study in the USA involving 108 patients with chronic medical conditions including cancers, it was found that a good working alliance between patients and the healthcare providers was strongly associated with patients’ adherence to and satisfaction with treatment [65]. A meta- analysis also showed that a good working alliance fosters adherence, satisfaction and improved patient outcomes [66]. In general, trust between patients and the healthcare providers promotes adherence. A review of 45 studies revealed that patients’ trust is likely enhanced by perceived physician’s technical competence, honesty, and willingness to involve patients in care. Patients are more likely to adhere to treatment plans when they perceive their healthcare providers as trusted [67]. In this regard, the UCI could further improve on the patient-clinician relationship in order to promote prompt health-seeking for cancers and adherence to cancer specific treatments at the facility.

Patients need to understand their conditions to help them anticipate the future. They need adequate and truthful information in order to facilitate psychological adjustments to their illnesses and treatments. Perceived truthfulness and details of information provided to patients and families are an important aspect of a meaningful therapeutic alliance. In a USA study among 305 colorectal cancer patients, it was found that open physician-patient engagement in communication was an important predictor of long term adherence to treatment and follow up care [68]. Communication was facilitated when patients perceived a trusting relationship between them and physicians [67]. While we were not able by the nature of our study design to determine predictors of adherence, our findings has very important implications for informing organization of patient-centered care service provisions at the UCI. In order to adequately inform service organization at the UCI and other similar centers, we do recommend a quantitative study to establish the magnitude of the perceived information inadequacy among the cancer patients as well as establish the characteristics of the healthcare providers reported to often provide inadequate information and or refuse to respond to patients’ questions.

### Limitations

This study has some limitations inherent in the design, approach and site. While the qualitative design allowed us to understand in details concerns regarding health-seeking and adherence to treatment, we could not establish magnitudes of associations and characteristics of the participants likely to be associated with the concepts explored. Second, the site for the study is the main national referral, specialized and training facility for cancers in Uganda. Therefore, transferability of findings from this study to cancer treatment facilities of lower levels need to carefully take this into considerations. In addition, the patients who have reached this referral facility are probably different in important ways from other esophageal cancer patients who have not managed to reach the facility for various reasons. Third, we sampled patients aged 40 years and above because they were the majority in the treatment center. However, this decision excluded the views of the younger patients who though few could have unique challenges to add to the debate.

## Conclusions

Health system and individual patient factors influence health-seeking for symptoms of esophageal cancer and adherence to treatment schedule for the disease. Increasing awareness about esophageal cancer risk factors and symptoms to the population so that they can determine perceived self-risk and attribute symptoms suggestive of the disease appropriately, and therefore promptly seek healthcare can lead to early detection of esophageal cancer. In addition, in-service training of primary healthcare professionals to improve their diagnostic acumens for esophageal and other cancers could minimize delay in cancer detection and avoid wasteful use of resources in managing cancer symptoms as other diseases at the lower level healthcare facilities.

## Supplementary Information

**Additional file 1.** In-depth Interview (IDI) Guide for patients with esophageal cancer

**Additional file 2.** Key Informant Interview (KII) Guide for the healthcare professionals

## Data Availability

The datasets used and/or analyzed during the current study are not publicly available because of ethical concerns regarding privacy and confidentiality of respondents but are available from the corresponding author on reasonable request.

## Abbreviations

AIDS: Acquired Immunodeficiency Syndrome
GERD: Gastro-esophageal reflux disorders
HIV: Human Immunodeficiency Virus
IDI: In-depth interview
KI: Key informant
KII: Key informant interview
LMICs: Low- and Middle-income countries
PUD: Peptic Ulcers Disease
UCI: Uganda Cancer Institute.
