# Supplementary material for: Understanding health-seeking and adherence to treatment by patients with esophageal cancer at the Uganda cancer Institute: a qualitative study
Source: BMC Health Serv Res. 2021 Feb 18;21:159. doi: 10.1186/s12913-021-06163-3 (PMC7890846; doi:10.1186/s12913-021-06163-3)
Supplement: Supplementary file 1 — Additional file 1. In-depth Interview (IDI) Guide for patients with esophageal cancer [file 12913_2021_6163_MOESM1_ESM.docx]

# In-depth Interview (IDI) Guide for patients with esophageal cancer

**Title: Perceived enablers and barriers to use of healthcare services among esophageal cancer patients at the Uganda cancer institute (UCI), Kampala.**

# General objective

To understand the perceptions and beliefs of patients with esophageal cancers regarding causes, symptoms and factors that enable and hinder utilization of healthcare services at the Uganda cancer institute, Kampala.

# Specific objectives

1. To explore the perceptions about help seeking for symptoms of esophageal cancer among patients with esophageal cancer attending care at the Uganda Cancer Institute.

2. To examine the perceived factors that influence access to and remaining in cancer specialized care among patients with esophageal cancer attending care at the Uganda Cancer Institute.

3. To understand the perspectives of the healthcare providers at the Uganda Cancer Institute regarding the challenges to health seeking and adherence to cancer specific treatment at the Uganda Cancer Institute.

**Participants:** Esophageal cancer patients attending care at the UCI.

**Time:** The interview is expected to last 30-50 minutes.

**Informed consent:** Purpose of the interview will be explained to participants and signed informed consent sought before interview.

**Venue:** A quiet room or open space to ensure openness and avoid interferences.

**Recordings:** Audio recordings will be done to augment field notes.

**Introduction:** In this study, we would like to understand the perceptions and beliefs of patients with esophageal cancers and healthcare professionals working with them regarding factors that enable and hinder utilization of healthcare services. Emphasis shall be put on the processes of health seeking and adherence to cancer specific treatment at the Uganda cancer institute, Kampala. In addition, we would also like to know how factors within the health system both at the UCI and before reaching the UCI influence the use of health services by patients with esophageal cancer. In particular, we would like to know whether and how distance to the UCI has influenced use of health services at the UCI by patients with esophageal cancer. Data generated from this study is expected to inform interventions to increase uptake of essential and specialized cancer services by patients with esophageal cancers; this could potentially lead to improvement in service delivery to patients with esophageal cancer, and lead to down-staging and better treatment outcomes from the cancer.

# Theme 1: Perceptions and beliefs about esophageal cancer causes and symptoms

1. Kindly tell me the things you consider or know to cause esophageal cancers.

**Probe:** Knowledge about esophageal cancer causes, risk factors, people at high risk for the cancer.

- 1. *What are the causes of esophageal cancer?*
  2. *Who are the people at higher risk for getting esophageal cancer?*

2. I would like to know how someone with esophageal cancer presents, i.e. the symptoms and signs of the cancer.

**Probes:** *What are the signs and symptoms of esophageal cancer?*

3. Kindly share with me your experience with esophageal cancer?

**Probes:**

*a) How did the symptoms of the disease now known to be esophageal cancer start in you; what did you first experience?*

*b) Which signs and symptoms did you experience?*

*c) When you developed those symptoms and signs, what did you first think was/were causing these symptoms?*

**Theme 2: Help seeking for symptoms**

1. Please tell me/us, response/reactions when you realized your symptoms were becoming serious.

**Probes:**

*a) What did you immediately do to deal with these symptoms?*

*b) Lay consultations for symptoms*

*(i) Tell me, did you tell anyone about your problem?*

*(ii) Who were these people (relationship with patient)?*

*(iii) What advice did they give you regarding the disease?*

*c) From the time when symptoms first started, how long did you take to visit a health facility because of these symptoms? Why did you take that amount of time before you visited a health facility? (Lack of money, no transport, waiting for an attendant etc).*

*d) Did you visit tradition health practitioners for your symptoms?*

*If yes, discuss why you consider visiting the traditional health practitioners.*

*If you did not visit any traditional health practitioners, please tell me why you did not do so.*

**Theme 3: Significant others in the pathway to treatment**

1 .Please tell us the people who supported you in this illness.

**Probes:**

*a) Did you tell anyone about your symptoms? Who? Why that person? What was his /her reaction?*

*b) Who influenced the decision to seek care in the health facility? How did they influence your decision?*

*c) Kindly share with me the different kind of support these different people provided to you since when you developed this illness.*

**Theme 4: Awareness and reaction to cancer diagnosis**

1. We would like to know what came to you mind when you were first told that your illness is due to a cancer of the throat.

**Probes:**

*a) When did you begin to suspect it was cancer?(Use calendar landmarks e.g. Easter, Christmas, Independence day, presidential election date etc. Please obtain month of event if patient cannot remember actual dates.*

*b) How did you feel about the possibility of having cancer? (Did you feel angry, indifferent, etc).*

*c) When did you confirm it was cancer?*

*d) Were there any barriers or challenges you faced while trying to confirm that you had cancer?*

**Theme 5: Histologic diagnosis and seeking for treatment**

1. Tell me about your experience during the course of diagnosis and treatment?

**Probes: (patient experience and health system factors)**

*a) How long did it take for you to decide to come for treatment after diagnosis?*

*b) Who influenced that decision?*

*c) What are the different types of treatments you have got so far for your cancer?*

*d) Were you involved in the decision of your treatment? How were you involved?*

*e) Were you given information about the treatment before they gave it you? In particular, please tell me/us the exact information you were given regarding the treatments?*

**Theme 6: Relationships and perceptions about the health system and staff**

1. Kindly tell me about your relationship with the healthcare givers at the UCI.

a) *How do you feel about the way they have been working with you?*

b) *Are there any issues you have against them – if yes, what are these issues?*

**Theme 7: Effect of illness and perceived quality of life**

1. Kindly share with me how you generally feel about your life since the diagnosis of this illness. **Probes:**

1. *How is your employment life now that you are sick?*
2. *Tell me about the effects of the disease and treatment on your body*
3. *Would you recommend someone else to seek care for esophageal cancer? And why?*
4. *Do you pay for the services? If so who helps you with the payments? Do you get difficulties in paying for the services?*

**Theme 8: Challenges and barriers to health seeking for esophageal cancer**

1. Please share with me/us some of the challenges and barriers you encounter before seeking care and while using healthcare services at this hospital?

***Probes:***

1. *Difficulty in getting to the right places (navigation)*
2. *Distance from home to the facility*
3. *Cost of transport and services*
4. *Fear of losing employment*
5. *Previous experience*
6. *Fear of diagnosis*
7. *Fear of treatment side effects*
8. *Lack of someone to keep home and care taker at the hospital*
9. *Inadequate information*
10. *Non recognition of symptom seriousness*
11. *Delayed referral by primary healthcare providers*
12. *Trying other alternatives like prayers, traditional medicine*
13. *Provider attitude*
14. *Did anyone make you feel bad about having esophageal cancer at any point?*
15. *Would you freely interact with other people or you could hide because of the disease?*
16. *How did you feel about how long it took you to begin treatment after you came to UCI?*
17. *Did you think it was too long before you were able to get treatment?*
18. What other factors may hinder a person with esophageal cancer from coming to seek care?

*Probe* *for myths and misconceptions about the disease.*

Thank you for your cooperation

| **Social demographic characteristics of participants** | | |
| --- | --- | --- |
| S.no | Question | Response |
| 1 | What is your age?  What is your date of birth? | ………………………..  …….............................. |
| 2 | What is your marital status? | 1. Married 2. Single 3. Divorced 4. Widow |
| 3 | What is your religion? | 1. Catholic 2. Anglican 3. Moslem 4. Others (specify)………. |
| 4 | What is your tribe? | ………………… |
| 5 | What is your Level of education? | 1. None 2. Primary 3. Secondary 4. Tertiary 5. University |
| 6 | What is your spouses’ level of education? | 1. None 2. Primary 3. Secondary 4. Tertiary 5. University |
| 7 | What is your occupation? | ………………………… |
| 8 | What is your estimated monthly house hold income | ………………………… |
| 9 | Residence | 1. Rural 2. Urban |
| 10 | What region of Uganda do you come from? | ……………………… |

*Thank you for your participation in the discussion*
