# Supplementary material for: Understanding health-seeking and adherence to treatment by patients with esophageal cancer at the Uganda cancer Institute: a qualitative study
Source: BMC Health Serv Res. 2021 Feb 18;21:159. doi: 10.1186/s12913-021-06163-3 (PMC7890846; doi:10.1186/s12913-021-06163-3)
Supplement: Supplementary file 2 — Additional file 2. Key Informant Interview (KII) Guide for the healthcare professionals [file 12913_2021_6163_MOESM2_ESM.docx]

# Key Informant Interview (KII) Guide for the healthcare professionals

**Title: Perceived enablers and barriers to use of healthcare services among esophageal cancer patients at the Uganda cancer institute (UCI), Kampala.**

# General objective

To understand the perceptions and beliefs of patients with esophageal cancers regarding causes, symptoms and factors that enable and hinder utilization of healthcare services at the Uganda cancer institute, Kampala.

# Specific objectives

1. To explore the perceptions about help seeking for symptoms of esophageal cancer among patients with esophageal cancer attending care at the Uganda Cancer Institute.

2. To examine the perceived factors that influence access to and remaining in cancer specialized care among patients with esophageal cancer attending care at the Uganda Cancer Institute.

3. To understand the perspectives of the healthcare providers at the Uganda Cancer Institute regarding the challenges to health seeking and adherence to cancer specific treatment at the Uganda Cancer Institute.

**Participants:** Healthcare professionals providing care to patients with esophageal cancer at the UCI.

**Time:** The interview is expected to last 30-50 minutes.

**Informed consent:** Purpose of the interview will be explained to participants and signed informed consent sought before interview.

**Venue:** A quiet room or open space to avoid interferences and ensure openness.

**Recordings:** Audio recordings will be done to augment field notes.

**Introduction:** In this study, we would like to understand the perceptions and beliefs of patients with esophageal cancers and healthcare professionals working with them regarding factors that enable and hinder utilization of healthcare services. Emphasis shall be put on the processes of health seeking and adherence to cancer specific treatment at the Uganda cancer institute, Kampala. In addition, we would also like to know how factors within the health system both at the UCI and before reaching the UCI influence the use of health services by patients with esophageal cancer. In particular, we would like to know whether and how distance to the UCI has influenced use of health services at the UCI by patients with esophageal cancer. Data generated from this study is expected to inform interventions to increase uptake of essential and specialized cancer services by patients with esophageal cancers; this could potentially lead to improvement in service delivery to patients with esophageal cancer, and lead to down-staging and better treatment outcomes from the cancer.

**Thematic area 1**

1. In what ways have you been involved in the esophageal cancer healthcare service delivery?

2. In your opinion what are the main reasons why esophageal patients present late and/or abscond from treatment?

**Probes**

1. *Non recognition of symptom seriousness*
2. *Delayed referral by primary healthcare providers*
3. *Distance to health facilities*
4. *Difficulty in getting to the right places (navigation)*
5. *Cost of transport and services*
6. *Inadequate information*

**Thematic area 2**

1. What experiences do patients go through during the course of their illness?

1. *Stress*
2. *Stigma*
3. *Loss of employment*
4. *Experiences related to the body*

2. What individual attributes facilitate the usage of esophageal cancer healthcare services?

**Probes:** Explore about: -

- - 1. *Age*
    2. *Marital status*
    3. *Education attainment*
    4. *Income*
    5. *Trust for health workers*
    6. *Trying other alternatives like prayers, traditional medicine*
    7. *Lack of someone to keep home and care taker at the hospital*
    8. *Patients’ attribution of symptoms and perceptions*

3. Which esophageal cancer characteristics contribute to healthcare services usage by the patients ***Probes: Inquire on: -***

*a) Stage at diagnosis*

1. *Symptom burden and fears regarding cancer treatment modalities and their perceived side effects*

4. What suggestions would you have for improving the delivery of healthcare services to this patient population?

***Thank you so very much for your participation; all your response are important to us.***
